# The Effect of Prophylactic Hepatoprotective Therapy on Drug-Induced Liver Injury in Patients Undergoing Chemotherapy for Cervical Cancer: A Retrospective Analysis Based on Propensity Score Matching

**DOI:** 10.3390/curroncol32070393

**Published:** 2025-07-09

**Authors:** Zhe Liu, Dongliang Yuan, Jun Chang, Lei Shi, Jingmeng Li, Mei Zhao, Qi Yang

**Affiliations:** 1 Department of Pharmacy, Xi’an Daxing Hospital, Yan’an University, Xi’an 710082, China; lz1272031654@163.com (Z.L.); lijingmeng88888888@163.com (J.L.); 2 Department of Pharmacy, Tangdu Hospital, Air Force Medical University, Xi’an 710038, China; dlyuantd@126.com (D.Y.); lanhai005@163.com (J.C.); shilei1860@126.com (L.S.)

**Keywords:** hepatoprotective drugs, drug-induced liver injury, cervical cancer, chemotherapy, propensity score matching

## Abstract

This retrospective study aimed to assess the effectiveness of prophylactic hepatoprotective therapy in decreasing the incidence of drug-induced liver injury (DILI) among patients with cervical cancer undergoing chemotherapy. The analysis was performed on patients with cervical cancer who received chemotherapy at a tertiary hospital between September 2019 and August 2020. Propensity score matching (PSM) was utilized to equilibrate baseline characteristics between the treatment group, which received prophylactic hepatoprotective drugs, and the control group, which did not receive prophylaxis. The incidence and severity of liver injury were evaluated using the Common Terminology Criteria for Adverse Events (CTCAE) version 5.0. Out of the 609 patients initially screened, 299 were included following PSM, with 105 in the treatment group and 194 in the control group. There were no significant differences in the incidence of liver injury (21.90% vs. 18.04%, *p* = 0.420) or its severity (*p* = 0.348) observed between the groups. Furthermore, none of the subgroups exhibited a significant reduction in DILI risk with prophylaxis. However, the number of patients experiencing an increase in their grade of liver injury was significantly higher in the treatment group (18.10% vs. 13.40%, *p* = 0.002), with these patients also exhibiting increased levels of alkaline phosphatase (ALP) and direct bilirubin (DBIL) post-chemotherapy (*p* < 0.05). Hepatoprotective drugs are not associated with a reduced risk of DILI and may in fact increase risk.

## Simple Summary

We investigated whether giving preventive liver-protecting drugs to cervical cancer patients before chemotherapy lowers their risk of treatment-related liver damage. We compared two carefully matched groups: one receiving these drugs and one not. We found that the preventive drugs did not reduce the chance of liver damage occurring, nor did they lessen its severity. Surprisingly, patients given the preventive drugs were more likely to experience worsening liver injury during treatment. These patients also showed higher levels of certain substances in their blood that indicate liver stress after chemotherapy. Our results suggest that routinely using these drugs to prevent liver injury in cervical cancer chemotherapy may not be beneficial and could potentially increase risks for some patients.

## 1. Introduction

Drug-induced liver injury (DILI) denotes hepatic damage resulting from exposure to drugs, encompassing both regulated therapeutic agents (prescription pharmaceuticals, OTC medications, biologics, standardized botanical preparations) and unregulated products (herbal remedies, nutraceuticals, dietary supplements) [1]. The incidence of DILI in the general population of mainland China is approximately 23.80 per 100,000 people, which is higher than the incidence reported in Western countries [2]. Antitumor drugs are an important etiology of DILI, with 8.34% of DILI cases in China being caused by antitumor drugs [3]. Studies have shown that antitumor drugs are the fifth most common cause of DILI in China [4].

Cervical cancer is a common malignant tumor in women, with 109,700 new cases and 59,100 deaths in China in 2020; this accounts for nearly 18% of the global morbidity and mortality rates [5]. Chemotherapy is one of the primary means of treating cervical cancer. However, most chemotherapeutic agents are hepatotoxic, which can cause DILI through different pathways and last for a long period, leading to the interruption of chemotherapy and affecting the efficacy of chemotherapy [6,7].

To avoid chemotherapy-induced DILI, clinicians usually administer hepatoprotective drugs to patients prophylactically, but evidence regarding the preventive use of hepatoprotective drugs and their ability to reduce the occurrence of chemotherapy DILI is not yet sufficient. In addition, the excessive use of hepatoprotective drugs may increase the economic burden of patients and cause other adverse drug reactions. Therefore, this study used a retrospective analysis to compare the occurrence of liver injury after chemotherapy in patients with cervical cancer who were and were not administered hepatoprotective drugs for prophylactic use, and aimed to explore the clinical significance of prophylactic hepatoprotective therapy.

## 2. Materials and Methods

### 2.1. Study Design and Ethical Approval

This retrospective study aimed to evaluate the effectiveness of hepatoprotective drugs in preventing DILI among patients with cervical cancer receiving chemotherapy. This research was approved by the Ethics Committee of Tangdu Hospital-Air Force Medical University (Protocol code: K202504-16). The need for individual patient consent was waived by the Committee due to the retrospective nature of the study.

### 2.2. Study Subjects

Data were collected from patients who were diagnosed with cervical cancer and received chemotherapy in a tertiary hospital between September 2019 and August 2020. These data included basic clinical data, liver function biochemical indexes, and data regarding the application of liver-protecting drugs, the application of antitumor drugs, and the application of a combination of drugs. The criteria for inclusion were as follows: (1) patients diagnosed with cervical cancer and receiving chemotherapy and (2) patients with a liver function index that was basically normal before chemotherapy. The criteria for exclusion were as follows: (1) patients with no liver function index test before and/or after chemotherapy; (2) patients with a combination of cholangiocarcinoma and primary or metastatic hepatocellular carcinoma; (3) patients with viral hepatitis; (4) patients with a combination of chronic basic diseases of the heart, liver, kidney, and other important organs, and hematopoietic dysfunction; (5) patients using a combination of medicines and with obvious liver damage (including but not limited to anti-tuberculosis drugs, NSAIDs, anti-HIV drugs, and Traditional Chinese Medicines); and (6) patients with a history of severe allergies, alcoholism, and drug addiction. Eligible patients were included in the control group (no prophylactic use of hepatoprotective drugs) and treatment group (prophylactic use of hepatoprotective drugs). The variety of hepatoprotective drugs and the mode of administration were chosen by the clinician, and the drugs were administered in the usual doses.

### 2.3. Observation Indicators

The results of the most recent liver function indexes before and after chemotherapy were recorded in both groups; these indexes included alanine aminotransferase (ALT), aspartate aminotransferase (AST), total bilirubin (TBIL), direct bilirubin (DBIL), ALP, and IBIL. The grade of hepatic damage was evaluated with reference to the National Cancer Institute (NCI)-released version 5.0 of the Common Terminology Criteria for Adverse Events (CTCAE) in 2017 [8]. Among these indicators, the highest toxicity classification rating was taken as the patient’s grade of hepatic damage. In this study, the upper limit of normal (ULN) was 40 U/L for AST and ALT, 135 U/L for ALP, and 17.1 μmol/L for TBIL.

### 2.4. Confounding Factor Treatment

Propensity score matching (PSM) is effective in minimizing confounding effects and balancing differences between groups in non-randomized clinical trials. In this study, the nearest neighbor matching method was utilized to match eligible patients 1:2, with the caliper value set at 0.01. The matching variables included the following: age, BMI, chemotherapy regimen (single-agent chemotherapy or multi-agent chemotherapy), and initial liver function indicators.

### 2.5. Statistical Analysis

SPSS software (Windows version 27.0, IBM Corp., Armonk, NY, USA) was used to perform statistical analyses. Measurements are expressed as the mean (±standard deviation) values and were analyzed using the independent samples *t*-test if they satisfied a normal distribution with chi-square. Otherwise, they are expressed as medians and quartiles and were analyzed using the Mann–Whitney U test. Count data are expressed as frequencies and rates and were analyzed using the chi-square test. All the hypothesis tests were two-sided, and differences were considered statistically significant at *p* value < 0.05.

### 2.6. Hepatoprotective Regimens

Hepatoprotective agent names, doses, and frequencies were extracted from the hospital information system. Hepatoprotective agents are used by doctors as appropriate before or on the day of chemotherapy based on the patient’s condition. All agents were classified into three mechanistic categories: glycyrrhizinates, Antioxidants, and phospholipids. Specific agents, doses, and frequencies are detailed in Table 1.

## 3. Results

### 3.1. Baseline Characteristics

**Figure 1:**
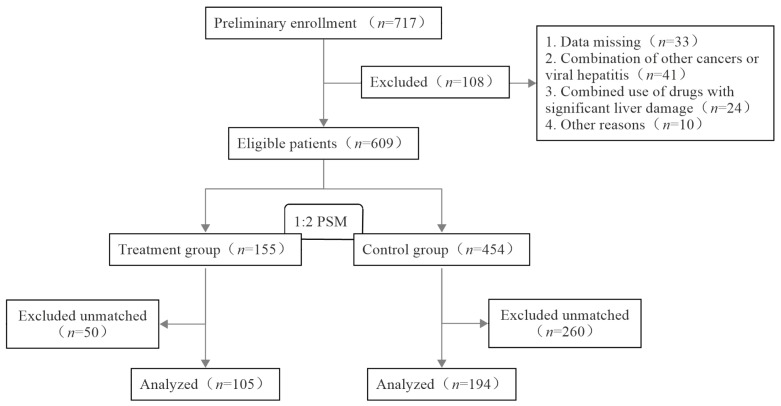
Flowchart of the case inclusion process.

A total of 609 patients were included in this study, with 155 in the treatment group and 454 in the control group. Before matching, the differences in the baseline age, BMI, chemotherapy regimen, ALT, AST, DBIL, and IBIL of the two groups were statistically significant (*p* < 0.05). To balance the baseline differences, 299 patients were finally included after PSM; this included 105 patients in the treatment group and 194 patients in the control group. The case inclusion process is shown in Figure 1. After matching, the differences in the baseline age, BMI, chemotherapeutic regimen, and pre-chemotherapeutic indicators of liver function between the two groups were not statistically significant (*p* > 0.05), as shown in Table 2.

### 3.2. Incidence of Liver Injury

Table 3 presents the incidence of liver injury after chemotherapy in the treatment and control groups. The prophylactic use of hepatoprotective drugs did not reduce the incidence of liver injury in chemotherapy patients.

### 3.3. Severity of Liver Injury

According to the NCI liver injury grading criteria, there was no statistically significant difference between the two groups in terms of the severity of liver injury after chemotherapy (Table 4). In the treatment group, 18.10% (19/105) of the patients saw an at least one-grade increase in their grade of liver injury, in comparison to 13.40% (26/194) in the control group. The difference between the two groups was statistically significant, as shown in Table 5.

### 3.4. Comparison of Liver Function Indicators

**Figure 2:**
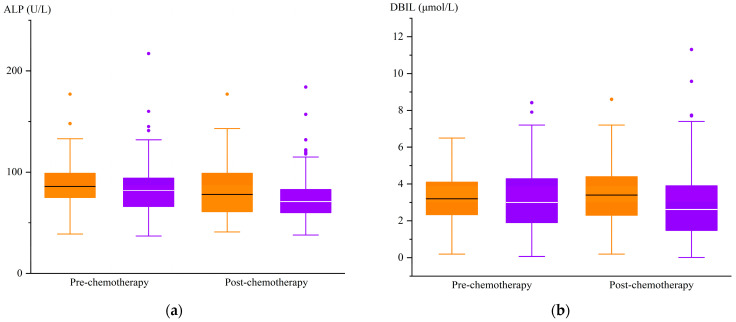
Changes in ALP and DBIL levels before and after chemotherapy. (**a**) 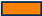, treatment group ALP; 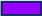, control group ALP. (**b**) 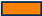, treatment group DBIL; 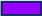, control group DBIL.

After chemotherapy, the treatment group exhibited higher ALP and DBIL levels compared to the control group (*p* < 0.05), and there was no difference in the levels of the other liver function indexes between the two groups (Table 6). The changes in the ALP and DBIL indexes of the two groups are shown in Figure 2.

### 3.5. Stratified Efficacy Analyses: Hepatoprotective Agents and Chemotherapy Regimens

#### 3.5.1. Prophylaxis Strategy: Monotherapy vs. Combination vs. Control

As shown in Table 7, neither monotherapy nor combination therapy significantly reduced risk compared to no prophylaxis (20.48% vs. 27.27% vs. 18.04%, *p* = 0.526). Notably, combination therapy demonstrated a numerically higher DILI rate than monotherapy (6.79% absolute increase).

#### 3.5.2. Subgroup Analysis by Monotherapy

Among monotherapy recipients, Glycyrrhizinates showed the highest DILI rate (24.2%, 16/66), followed by polyene phosphatidylcholine (10.0%, 1/10) and glutathione (0%, 0/7) (Table 8). However, no significant between-group differences were detected (*p* = 0.446) due to the limited sample size in nonglycyrrhizinate groups.

#### 3.5.3. Subgroup Analysis by Chemotherapy Regimen

The distribution of chemotherapy regimens is summarized in Table 9. Given the dominance of platinum-based regimens (86.63%, 259/299), we focused subsequent analyses on three major categories: platinum monotherapy (29.77%), platinum–taxane combinations (48.16%), and platinum–gemcitabine (8.70%).

In platinum–taxane regimens (Table 10), glycyrrhizinate monotherapy showed comparable DILI risk to the control group (17.24% vs. 18.18%, *p* > 0.05). Combination therapy demonstrated a numerically elevated DILI rate (30.00% vs. 18.18%, *p* > 0.05).

For other platinum-based regimens (Table 11), descriptive analyses were performed due to the limited sample size per subgroup (*n* ≤ 11). Notably, platinum–gemcitabine regimens demonstrated consistently elevated DILI rates (33.33–66.67%), with substantially numerically higher rates than both platinum monotherapy (9.09–25.00%) and platinum–taxane regimens (17.24–30.00%).

## 4. Discussion

Almost all antitumor drugs exhibit hepatotoxicity to some extent, with liver injury being a common adverse event following the administration of antitumor drugs [9,10]. In China, clinicians usually give patients prophylactic hepatoprotective treatment to avoid chemotherapy-induced liver injury. However, our study found that prophylactic hepatoprotective therapy had no significant beneficial effect. Specifically, the treatment group showed no improvement in the incidence, severity, or grade progression of liver injury, nor was there an improvement in the liver function indexes, compared to the control group. Notably, the treatment group exhibited higher post-chemotherapy ALP and DBIL levels (*p* < 0.05), and patients in this group were more likely to experience an increase in their grade of liver injury (18.10% vs. 13.40%, *p* = 0.002). Furthermore, only 3.81% (4/105) of patients in the treatment group experienced a reduction in their grade of liver injury, compared to 13.40% (26/194) in the control group. These findings challenge the rationale for routine prophylactic hepatoprotective therapy, as it neither prevented DILI nor preserved baseline liver function.

The analysis of hepatoprotective strategies revealed that monotherapy exhibited a DILI rate (20.48%) comparable to the control group (18.04%), with combination therapy also failing to reduce risk significantly (Table 7). Among monotherapy subgroups, DILI rates varied numerically across agents (glycyrrhizinates/glutathione/phospholipids) but without statistical significance (Table 8). Focusing on platinum-based regimens (86.63%, 259/299), we excluded undersampled regimens (e.g., platinum–etoposide, *n* = 2) to avoid bias from low statistical power. No subgroup demonstrated significant DILI reduction with prophylaxis across chemotherapy categories. For platinum monotherapy and platinum–gemcitabine, descriptive analyses were performed without statistical metrics due to limited subgroup samples (e.g., glutathione *n* = 0) (Table 11). Notably, platinum–gemcitabine regimens showed systematically elevated DILI incidence (33.33–66.67%), exceeding platinum–taxane regimens (17.24–30.00%) by >3-fold, though cautious interpretation is warranted given sample constraints (Table 10 and Table 11). This divergence may stem from gemcitabine’s unique hepatotoxicity profile, inducing sinusoidal obstruction syndrome [6,11]. Collectively, hepatoprotective drugs did not reduce DILI risk in this cohort, with certain strategies potentially increasing risk.

While this cohort study failed to demonstrate a significant protective effect of prophylactic hepatoprotective agents against chemotherapy-associated drug-induced liver injury, the potential value of specific agents in the context of precision medicine warrants attention. In anti-tuberculosis therapy, for instance, evidence supports the hepatoprotective effect of silymarin against short-term liver injury. A meta-analysis revealed a significant reduction in DILI risk at week 4 of treatment [RR: 0.33, 95% CI (0.15, 0.75)]. However, this protective effect was strictly time-dependent and was not sustained over longer treatment durations [12]. More critically, a multicenter randomized trial (*n* = 568) in patients with tuberculosis without high-risk factors demonstrated that silymarin failed to reduce the incidence of DILI (silymarin group: 12.6% vs. control group: 14.1%, *p* = 0.32) [13]. Consequently, our findings, combined with the existing literature, suggest that DILI prevention strategies in chemotherapy patients should draw lessons from tuberculosis management. Namely, given the potential mechanistic differences in DILI of varying etiologies [14,15], mechanism-guided precision prophylaxis targeting specific high-risk subgroups (e.g., patients with baseline liver function abnormalities, receiving specific chemotherapy regimens, or harboring genetic polymorphisms) should be prioritized over routine universal administration.

Age, female sex, race, alcoholism, viral hepatitis, anti-HIV treatment, and anti-tuberculosis treatment were risk factors for DILI [16]. To address confounding variables, this study excluded high-risk populations and incorporated age, BMI, chemotherapy regimen, and baseline liver function into the propensity score matching model. Studies have shown that the occurrence of DILI is also closely related to the daily dose, lipophilicity, and degree of drug metabolism. Drugs with daily doses higher than 50~100 mg per day and higher lipophilicity are more likely to cause DILI. Lipophilic drugs are distributed in many different tissues and organs and need to be converted to hydrophilic metabolites to be eliminated; this requires more metabolites to be excreted from the body, which may increase the risk of liver injury [16,17,18,19,20,21]. Therefore, more-lipophilic hepatoprotective drugs such as silymarins and polyene phosphatidylcholine may also increase the risk of hepatic damage in chemotherapy patients; this could explain the greater increase in the grade of hepatic damage in patients in the treatment group. In addition, liver-protecting drugs are medications that need to be metabolized by the liver. For chemotherapy patients, the prophylactic use, heavy use, or abuse of hepatoprotective drugs may increase the burden on the liver, particularly when they are at risk of hepatic damage caused by antitumor drugs.

An increased risk of hepatic damage could be associated with the use of hepatoprotective agents or chronic hepatitis B infection [22]. Supporting this hypothesis, a randomized controlled trial evaluating the use of silymarin to prevent tuberculosis-related DILI similarly found that it had no protective effect; the study even noted that it increased the risk of liver injury [23]. The results of the present study are also similar to the results of this randomized controlled trial, in which the prophylactic use of hepatoprotective drugs did not significantly reduce the incidence of hepatic damage, but may have made liver injury more severe. It has been reported that magnesium isoglycyrrhizinate can worsen liver injury [24]. In addition, studies have shown that liver-protecting drugs do not shorten the time required for liver enzymes to return to normal, regardless of the severity of liver injury [25]. Unlike mainland China, a wide variety of hepatoprotective drugs is not available overseas, and it is thought that most DILIs can recover on their own without any treatment or specific measures. It has also been suggested that the timely discontinuation of the suspected drug is the most crucial step in the treatment of suspected DILI [14,15,16]. Therefore, clinicians should carefully decide whether to use hepatoprotective drugs to prevent DILI in chemotherapy patients.

## 5. Strengths and Limitations

There is very limited information on the use of prophylactic hepatoprotective agents in preventing DILI from various classes of agents. This study has explored an important area of drug-induced liver injury—namely, the use of prophylactic hepatoprotective agents to prevent chemotherapy-associated DILI. However, their protective effect could not be demonstrated in this study. Hepatoprotective drugs are not associated with a reduced risk of DILI and may in fact increase risk. Our findings may have sounded the alarm for the rational use of hepatoprotective agents. Furthermore, propensity score matching was used to balance the confounding effects in non-randomized clinical studies, making the results more reliable.

There are several limitations to this study. First, this is a single-center study, thus restricting generalizability. Second, in order to minimize the various confounding biases associated with the use of real-world data, the PSM method was used to match the data in this study, which resulted in the loss of a large portion of unsuccessfully matched data, leading to a reduced sample size. Third, because this study was a retrospective study, the timing of liver function testing in patients after chemotherapy was not uniform; certain patients were tested within one day after chemotherapy, while other patients were tested after the third day or more after chemotherapy. The different times at which the patients’ liver function indexes were measured may have had an impact on the test results. Fourth, this study was unable to obtain data outside of the hospital information system, and certain patients may have taken medications with hepatic injurious effects that were not recorded in the information system. Fifth, the retrospective design inherently introduced selection bias. Physicians may have preferentially prescribed hepatoprotective agents to patients perceived to be at higher risk (e.g., those with baseline liver dysfunction or intensive chemotherapy regimens), thus potentially inflating the observed DILI risk in the prophylaxis group. Moreover, the limited sample size reduced statistical power to detect clinically meaningful differences, particularly in subgroup analyses, where results should be interpreted with caution. Future large-scale prospective cohorts are warranted to validate these findings.

## 6. Conclusions

In this study, prophylactic hepatoprotective therapy did not show a significant positive effect on patients treated with chemotherapy for cervical cancer, and the grade of liver injury tended to increase in patients on prophylactic hepatoprotective drugs. Hepatoprotective drugs are not associated with a reduced risk of DILI and may in fact increase risk. Clinicians should be cautious in deciding whether or not to administer prophylactic hepatoprotective therapy to chemotherapy patients in order to ensure the safety of their medications.

## Figures and Tables

**Table 1:** Prophylactic hepatoprotective agents used in the study.

Category	Specific Agent	Cases	Typical Dose	Frequency
Glycyrrhizinates	Magnesium Isoglycyrrhizinate Injection	53	200 mg	QD ^#^
	Ammonium Glycyrrhizinate–Cysteine Injection	13	100 mL	QD
Antioxidants	Reduced Glutathione for Injection	7	10 mL	QD
Phospholipids	Polyene Phosphatidylcholine Injection	10	10 mL	QD
Combination Therapy	Glycyrrhizinate + Glutathione *	17	/	/
	Glycyrrhizinate + Phospholipid **	5	/	/

* Included 15 cases of magnesium isoglycyrrhizinate injection and 2 cases of Ammonium Glycyrrhizinate–Cysteine Injection. ** Included 4 cases of magnesium isoglycyrrhizinate injection and 1 case of Ammonium Glycyrrhizinate–Cysteine Injection. ^#^ QD: once a day.

**Table 2:** General characteristics of patients between the treatment and control groups.

General Characteristics	Before Propensity Score Matching			After Propensity Score Matching		
	Control Group (*n* = 454)	Treatment Group (*n* = 155)	*p* Value	Control Group (*n* = 194)	Treatment Group (*n* = 105)	*p*
Age (years)	53 (45, 58)	54 (49, 60)	0.015	53 (47, 60)	53 (49, 57)	0.952
BMI	22.8 (20.8, 25.2)	24 (21.5, 25.5)	0.008	23 (21.1, 25.5)	23.6 (21.4, 25.5)	0.643
Chemotherapy regimen *	220/234	47/108	<0.001	83/111	39/66	0.343
ALT (U/L)	23 (16, 30)	18 (12, 26)	<0.001	21 (15, 28)	20 (12, 28)	0.301
AST (U/L)	25 (21, 30)	22 (19, 27)	<0.001	23 (20, 28)	24 (21, 28)	0.885
ALP (U/L)	80 (65, 94)	86 (74, 101)	0.123	82 (66, 94)	86 (75, 99)	0.078
TBIL (μmol/L)	10.58 (8.10, 13.90)	10.10 (8.87, 12.60)	0.820	10.89 (8.48, 14.28)	9.90 (8.90, 12.80)	0.269
DBIL (μmol/L)	2.72 (1.60, 3.75)	3.40 (2.50, 4.40)	<0.001	3.00 (1.87, 4.29)	3.20 (2.32, 4.14)	0.266
IBIL (μmol/L)	7.70 (5.87, 10.45)	6.90 (5.60, 8.90)	0.007	7.82 (5.93, 10.25)	7.30 (6.00, 8.95)	0.103

* Number of patients using single-agent chemotherapy regimens or multi-agent chemotherapy regimens.

**Table 3:** Comparison of the incidence of liver injury between the treatment and control groups.

Group	Liver Injury		Incidence of Liver Injury	χ^2^	*p*
	No	Yes			
Control group (*n* = 194)	159	35	18.04%	0.650	0.420
Treatment group (*n* = 105)	82	23	21.90%		

**Table 4:** Comparison of NCI grading of severity of liver injury between the treatment and control groups.

Group	Grade 0	Grade 1	Grade 2	Grade 3	Grade 4	*U*	*p*
Control group (*n* = 194)	159	33	2	0	0	9725.000	0.348
Treatment group (*n* = 105)	82	18	3	2	0		

**Table 5:** Comparison of changes in NCI grading of liver injury after chemotherapy between treatment and control groups.

Group	Down 1 Level	Unchanged	Up 1 Level	Up 2 Levels	Up 3 Levels	*U*	*p*
Control group (*n* = 194)	26	8	26	0	0	490.000	0.002
Treatment group (*n* = 105)	4	4	14	3	2		

**Table 6:** Comparison of liver function indexes after chemotherapy between treatment and control groups.

Group	ALT (U/L)	AST (U/L)	ALP (U/L)	TBIL (μmol/L)	DBIL (μmol/L)	IBIL (μmol/L)
Control group (*n* = 194)	21 (16, 28)	25 (20, 31)	70 (60, 83)	11.58 (7.88, 14.83)	2.62 (1.48, 3.90)	8.20 (5.59, 11.56)
Treatment group (*n* = 105)	20 (13, 29)	24 (20, 31)	77 (60, 100)	11.10 (8.98, 13.81)	3.40 (2.30, 4.40)	7.70 (5.75, 9.90)
*U*	9233.500	10,082.000	4418.000	9867.000	7851.000	9682.000
*p*	0.182	0.885	0.038	0.656	0.001	0.481

**Table 7:** Comparison of the incidence of liver injury in different prophylaxis strategies.

Group	Liver Injury		Incidence of Liver Injury	Fisher Value *	*p*
	No	Yes			
Monotherapy (*n* = 83)	66	17	20.48%	1.359	0.526
Combination therapy (*n* = 22)	16	6	27.27%		
Control group (*n* = 194)	159	35	18.04%		

* Fisher’s exact test was used for small subgroups.

**Table 8:** Comparison of the incidence of liver injury in monotherapy.

Group	Liver Injury		Incidence of Liver Injury	Fisher Value	*p*
	No	Yes			
Glycyrrhizinates (*n* = 66)	50	16	24.24%	2.679	0.446
Glutathione (*n* = 7)	7	0	0.00%		
Polyene Phosphatidylcholine (*n* = 10)	9	1	10.00%		
Control group (*n* = 194)	159	35	18.04%		

**Table 9:** Distribution of chemotherapy regimens in the cohort.

Chemotherapy Regimen	Subgroup	Cases	Proportion
Platinum Monotherapy	/	89	29.77%
Platinum + Taxane	Platinum + Paclitaxel	72	24.08%
	Platinum + Docetaxel	72	24.08%
Platinum + Gemcitabine	/	26	8.70%
Other Regimens	/	40	13.38%

**Table 10:** DILI risk in platinum–taxane regimens by prophylaxis strategy.

Group	Liver Injury		Incidence of Liver Injury	Fisher Value *	*p*
	No	Yes			
Glycyrrhizinate monotherapy (*n* = 29)	24	5	17.24%	1.119	0.568
Glutathione monotherapy (*n* = 1)	1	0	0.00%		
Polyene phosphatidylcholine monotherapy (*n* = 5)	5	0	0.00%		
Combination therapy (*n* = 10)	7	3	30.00%		
Control group (*n* = 99)	81	18	18.18%		

* Fisher’s exact test was used for small subgroups; results with *n* ≤ 5 per cell are descriptive only.

**Table 11:** DILI risk in other chemotherapy regimens.

Chemotherapy Regimen	Prophylaxis Group	Liver Injury		Incidence of Liver Injury
		No	Yes	
Platinum Monotherapy	Glycyrrhizinate monotherapy	5	1	16.67%
	Glutathione monotherapy	0	0	/
	Polyene phosphatidylcholine monotherapy	2	0	0.00%
	Combination therapy	3	1	25.00%
	Control group	70	7	9.09%
Platinum + Gemcitabine	Glycyrrhizinate monotherapy	4	7	63.64%
	Glutathione monotherapy	0	0	/
	Polyene phosphatidylcholine monotherapy	0	0	/
	Combination therapy	4	2	33.33%
	Control group	3	6	66.67%

## Data Availability

Data is unavailable due to privacy or ethical restrictions.

## Abbreviations

The following abbreviations are used in this manuscript:

DILI	Drug-induced liver injury
PSM	Propensity score matching
CTCAE	Common Terminology Criteria for Adverse Events
ALP	Alkaline phosphatase
ALT	Alanine aminotransferase
AST	Aspartate aminotransferase
TBIL	Total bilirubin
DBIL	Direct bilirubin
NCI	National Cancer Institute
ULN	Upper limit of normal

## References

[1] Technology Committee on DILI Prevention and Management Chinese Medical Biotechnology Association, Study Group of Drug-Induced Liver Disease Chinese Medical Association for the Study of Liver Diseases (2023). Chinese guideline for diagnosis and management of drug-induced liver injury (2023 version). Zhonghua Gan Zang Bing Za Zhi.

[2] Shen T., Liu Y., Shang J., Xie Q., Li J., Yan M., Xu J., Niu J., Liu J., Watkins P.B. (2019). Incidence and Etiology of Drug-Induced Liver Injury in Mainland China. Gastroenterology.

[3] Li X., Tang J., Mao Y. (2022). Incidence and risk factors of drug-induced liver injury. Liver Int..

[4] Zhou Y., Yang L., Liao Z., He X., Zhou Y., Guo H. (2013). Epidemiology of drug-induced liver injury in China: A systematic analysis of the Chinese literature including 21,789 patients. Eur. J. Gastroenterol. Hepatol..

[5] Bruni L.A.G., Serrano B., Mena M., Collado J.J., Gómez D., Muñoz J., Bosch F.X., de Sanjosé S., ICO/IARC Information Centre on HPV and Cancer (HPV Information Centre) Human Papillomavirus and Related Diseases in China. https://hpvcentre.net/statistics/reports/CHN.pdf?t=1678773276663.

[6] Vincenzi B., Armento G., Spalato Ceruso M., Catania G., Leakos M., Santini D., Minotti G., Tonini G. (2016). Drug-induced hepatotoxicity in cancer patients-implication for treatment. Expert Opin. Drug Saf..

[7] Vigano L., De Rosa G., Toso C., Andres A., Ferrero A., Roth A., Sperti E., Majno P., Rubbia-Brandt L. (2017). Reversibility of chemotherapy-related liver injury. J. Hepatol..

[8] U.S. Department of Health and Human Services, National Institutes of Health, National Cancer Institute Common Terminology Criteria for Adverse Events (CTCAE) Version 5.0. https://ctep.cancer.gov/protocolDevelopment/electronic_applications/docs/CTCAE_v5_Quick_Reference_5x7.pdf.

[9] Jeong J., Park S., Heo K.N., Park S.M., Min S., Ah Y.M., Han J.M., Lee J.Y. (2024). Comprehensive analysis of nationwide anticancer drug-related complications in Korea: Incidence, types, and cancer-specific considerations in contemporary oncology. Ther. Adv. Med. Oncol..

[10] Atteia H.H. (2024). MicroRNAs in Anticancer Drugs Hepatotoxicity: From Pathogenic Mechanism and Early Diagnosis to Therapeutic Targeting by Natural Products. Curr. Pharm. Biotechnol..

[11] Hailan W.A.Q., Abou-Tarboush F.M., Al-Anazi K.M., Ahmad A., Qasem A., Farah M.A. (2020). Gemcitabine induced cytotoxicity, DNA damage and hepatic injury in laboratory mice. Drug Chem. Toxicol..

[12] Tao L., Qu X., Zhang Y., Song Y., Zhang S.X. (2019). Prophylactic Therapy of Silymarin (Milk Thistle) on Antituberculosis Drug-Induced Liver Injury: A Meta-Analysis of Randomized Controlled Trials. Can. J. Gastroenterol. Hepatol..

[13] Gu J.T.S., Tan S.Y., Wu Q., Zhang X., Liu C.X., Gao X.S., Yuan B.D., Han L.J. (2016). An open-label randomized and multi-center clinical trial to evaluate the efficacy of Silibinin in preventing drug-induced liver injury (In Chinese). Chin. J. Antituberc..

[14] Björnsson H.K., Björnsson E.S. (2022). Drug-induced liver injury: Pathogenesis, epidemiology, clinical features, and practical management. Eur. J. Intern. Med..

[15] Andrade R.J., Chalasani N., Björnsson E.S., Suzuki A., Kullak-Ublick G.A., Watkins P.B., Devarbhavi H., Merz M., Lucena M.I., Kaplowitz N. (2019). Drug-induced liver injury. Nat. Rev. Dis. Primers.

[16] (2019). European Association for the Study of the Liver EASL Clinical Practice Guidelines: Drug-induced liver injury. J. Hepatol..

[17] McEuen K., Borlak J., Tong W., Chen M. (2017). Associations of Drug Lipophilicity and Extent of Metabolism with Drug-Induced Liver Injury. Int. J. Mol. Sci..

[18] Chen M., Borlak J., Tong W. (2013). High lipophilicity and high daily dose of oral medications are associated with significant risk for drug-induced liver injury. Hepatology.

[19] Lammert C., Einarsson S., Saha C., Niklasson A., Bjornsson E., Chalasani N. (2008). Relationship between daily dose of oral medications and idiosyncratic drug-induced liver injury: Search for signals. Hepatology.

[20] Ortega-Alonso A., Stephens C., Lucena M.I., Andrade R.J. (2016). Case Characterization, Clinical Features and Risk Factors in Drug-Induced Liver Injury. Int. J. Mol. Sci..

[21] Kaplowitz N. (2013). Avoiding idiosyncratic DILI: Two is better than one. Hepatology.

[22] Shen X., Yuan Z., Mei J., Zhang Z., Guo J., Wu Z., Wu J., Zhang H., Pan J., Huang W. (2014). Anti-tuberculosis drug-induced liver injury in Shanghai: Validation of Hy’s Law. Drug Saf..

[23] Zhang S., Pan H., Peng X., Lu H., Fan H., Zheng X., Xu G., Wang M., Wang J. (2016). Preventive use of a hepatoprotectant against anti-tuberculosis drug-induced liver injury: A randomized controlled trial. J. Gastroenterol. Hepatol..

[24] Gu S.W.Z.B. (2012). Magnesium isoglycyrrhizinate-induced liver damage: A case report (In Chinese). Chin. J. Hepatol. (Electron Ed.).

[25] Saito Z., Kaneko Y., Kinoshita A., Kurita Y., Odashima K., Horikiri T., Yoshii Y., Seki A., Seki Y., Takeda H. (2016). Effectiveness of hepatoprotective drugs for anti-tuberculosis drug-induced hepatotoxicity: A retrospective analysis. BMC Infect. Dis..

